# Computational prediction of binding affinity and structural impact of three Pakistani SARS-CoV-2 spike RBD variants on human ACE2 interaction

**DOI:** 10.1371/journal.pone.0346242

**Published:** 2026-04-01

**Authors:** Muhammad Usama, Muhammad Azeem, Ghulam Mustafa

**Affiliations:** 1 Department of Biochemistry, Government College University Faisalabad, Faisalabad Pakistan; 2 College of Life Sciences, Anhui Normal University, Wuhu, China; University of the Witwatersrand, SOUTH AFRICA

## Abstract

Despite the current development and progress in vaccine synthesis, the transmission and infection rate of new variants of SARS-CoV-2 is still challenging in developing countries like Pakistan. Previous studies reported the transmission of new virus variants associated with the mutation in spike protein particularly in the receptor binding domain (RBD) of SARS-CoV-2 making RBD a promising target to control the infection rate. Any change in the spike RBD affects the interaction with host angiotensin-converting enzyme 2 (hACE2) receptor. These mutations in RBD assist SARS-CoV-2 entry in the host and enhance virulence and transmission rate. In the current study, we retrieved all substitution mutations in RBD of spike protein in three Pakistani SARS-CoV-2 variants from the GISAID database. Their structures were predicted via ColabFold2 and protein-protein docking was performed using HADDOCK. Interactions between docked complexes were checked by PyMOL and binding affinity (ΔG) (kcal/mol) was calculated via Prodigy server. The results of protein-protein interaction showed that the mutated hCoV-12471804 variant exhibited a higher number of interactions (202), low Z-score (−2.1), increased buried surface area (4066.4 ± 55.9), and high binding affinity (−21.3 kcal/mol) with hACE2 compared to hCoV-Wild variant. Moreover, the current study provides insights into the effects of mutations in RBD with hACE2 interaction providing deeper structural insight to understand the molecular mechanism responsible for the increased transmissibility of newly emerged SARS-CoV-2 variants. The exclusively in silico nature of this study necessitates experimental validation to definitively quantify the kinetic and thermodynamic parameters of the hACE2-RBD interaction.

## Introduction

The coronavirus disease also known as COVID-19 caused by SARS-CoV-2 in humans has been considered the worst infectious disease since its first report in mid-December 2019 in Wuhan China. Due to altered transmissibility and therapeutic resistance, COVID-19 emerged as the most severe pandemic [1]. Currently, COVID-19 cases have shown fluctuations globally, while emerging variants causing localized outbreak, while vaccination have helped to reduce severe illness and deaths. The COVID-19 pandemic has significantly squeezed developing countries like Pakistan and become the foremost challenge for the economy after multiple waves of infection [2]. Despite nationwide lockdown and travel restrictions, Pakistan faced several hurdles to tackle this pandemic due to the incomplete healthcare infrastructure and inadequate testing capabilities. Moreover, Pakistan and other developing countries continued to overcome the challenges and to steer the pandemic aftermath [3].

To understand the transmissibility of viral variants, it is essential to acknowledge how confined evolutionary pressures influenced the viral diversity. Pakistan faced multiple infection waves influenced by geographical factors, large population, and usage of different vaccines to overcome this pandemic [1]. These factors influenced the mutation patterns and adoptative behavior of the mutated variants. Therefore, it is necessary to shed light on regional dynamics that modulate the viral evolution and spread around the globe.

In 2020, researchers revealed that the Angiotensin-converting enzyme 2 (ACE2) facilitates the SARS-CoV to enter the human body by acting as a receptor [4]. Early research provided insights into how the spike protein of coronavirus recognized its receptor by providing the experimental structures of the SARS-CoV-2 RBD–ACE2 complex [5]. The human cell surface has angiotensin-converting enzyme 2 (ACE2) which facilitates the entry of SARS-CoV-2 virus by interacting with spike protein present on the surface of coronavirus. The membrane-distal region of the ACE2 interacts with the receptor-binding domain of the spike protein [6]. Shortly after synthesis, the homotrimer of the spike protein is divided into two subunits but remains connected non-covalently: S1, which contains the receptor-binding domain, and S2, which facilitates membrane fusion upon ACE2 binding [7].

Studies have shown that the receptor binding domain (RBD) of spike protein could be one of the major targets to combat this disease [8,9]. Over the last two years, it was observed that the level of IgG and IgM antibodies has been decreased against the receptor binding domain of spike protein whereas the IgA remained less affected [10]. At the same time, several studies have reported the mutations in spike protein which enabled the SARS-CoV-2 spike protein to escape from these neutralizing antibodies that raised concerns about the efficacy and potency of monoclonal antibodies as well as antiviral drugs and vaccines [11,12]. The mutations in the RBD of spike protein are making the pandemic harder by enhancing the viral transmissibility and resistance to natural protective immunity as well as escape from antiviral drugs [13].

The mutated SARS-CoV-2 variants are more contagious compared to the parent variant. These variants include alpha, beta, delta, gamma, and omicron and contain mutated surface spike protein which enables the virus to spread the infection [14]. The host susceptibility to COVID-19 infection has been determined by the affinity between receptor binding domain of spike protein and human ACE2 [14]. Although previous structural knowledge on the SARS-CoV–ACE2 binding complex exists, the mechanism by which mutations in the spike protein affect molecular recognition remains unclear. The effects of these mutations on protein dynamics, structure, and interaction with ACE2 are still not well addressed.

Nowadays, different computational tools and software such as molecular docking and molecular dynamics simulation are employed to study the effects of the mutations on protein function, structure, and molecular interactions at the atomic level. In the current investigation, three SARS-CoV-2 variants with high rate of amino acid substitution mutations, were selected and used to explore the accumulative effects of mutations in RBD of spike protein on binding with hACE2 receptor. The current study was aimed to provide a comparative understanding of newly emerging SARS-CoV-2 mutated variants by employing molecular docking and molecular dynamics simulations to evaluate the effects of accumulated mutations in the spike receptor-binding domain on binding affinity, structural stability, and conformational behavior upon interaction with the human ACE2 receptor, thereby supporting vaccine and therapeutic design.

## Materials and methods

### Retrieval of SARS-CoV-2 spike protein sequences

Spike protein sequences of SARS-CoV-2 were retrieved from GISAID database (https://gisaid.org/) (accessed on June 7, 2024) [15]. The SARS-CoV-2 spike protein sequence of reference genome hCoV-19/Wuhan/WIV04/2019 (hCoV-WT hereafter) at GISAID database with accession number (EPI_ISL_402124-S) was used as the wild type. The GISAID database was screened out by location using “Pakistan” as query to find the SARS-CoV-2 variants with high rate of amino acid substitutions. Analysis of 1236 Pakistani spike protein sequences reported between sampling window (February 2020 to May 2024) was assayed from GISAID database. The sequences of the wild type and three SARS-CoV-2 variants belong to Omicron lineage including hCoV-19/Pakistan/UOL-IMBB/2021–7 (hCoV-UOL-IMBB hereafter) (accession number: EPI_ISL_5411332) (Pango lineage: BA.5.2.14) observed in September 2022 (Prevalence 4.02%), hCoV-19/Pakistan/12431387/2022 (hCoV-12431387 hereafter) (accession number: EPI_ISL_15316452) (Pango lineage: XBB.19.1) observed from March to August, 2023, (Prevalence 8.01%) and hCoV-19/Pakistan/12471804/2022 (hCoV-12471804 hereafter) (accession number: EPI_ISL_15316448) (Pango lineage: XBB.1.19.1) observed from March to August, 2023, (Prevalence 9.06%) were retrieved from GISAID database for detailed analyses based on greater number of mutations in the receptor binding domain of the spike protein. The Clustal W tool in MEGA X software (version 10.0.5) was used for multiple sequence alignment analysis (MSA) to compare the mutated sequences with the wild one [16].

### Protein domain and homology-based structure prediction

InterPro database (https://www.ebi.ac.uk/interpro/) (accessed on June 11, 2024) was used to find the position of receptor binding domain (Pfam ID: PF09408 and InterPro ID: IPR018548) in the spike protein sequences of wild and mutant variants, to check the similarities and mutations in RBD among selected three viral variants. The 3D structure prediction was done by ColabFold2 with recycles count 3 and five number of predicted models (https://colab.research.google.com/github/sokrypton/ColabFold/blob/main/AlphaFold2.ipynb) [17]. The 3D structure of human angiotensin-converting enzyme 2 was retrieved from Protein Data Bank with PDB ID: 7V84 (accessed on June 13, 2024). The quality of the computationally predicted 3D structure of RBDs was confirmed by PDBSum online tool (accessed on June 15, 2024) [18]. UCSF Chimera software (version 1.16) was used for 3D structural visualization, comparison, and superimposition of RBD structures of wild and mutants to highlight the aligned regions [19].

### Protein-protein docking

The protein-protein docking to predict interactions between RBD od SARS-CoV-2 and human ACE2 was performed via HADDOCK server (https://rascar.science.uu.nl/haddock2.4/; accessed on August 5, 2024) in three independent runs, with the top-ranked model from each run selected for subsequent analyses. The molecular docking was carried out by selecting whole receptor binding domain via the HADDOCK server with default parameters to explore specific protein–protein interactions between human angiotensin-converting enzyme 2 (ACE2) and four SARS-CoV-2 viral spike RBDs variants [20]. The educational license of PyMOL Molecular Graphics System (version 2.5.4) was used to visualize and draw the interactions at 4.0 Å between the active interacting residues of ACE2 and spike RBDs from SARS-CoV-2 variants. The predicted values for binding affinity (ΔG) (kcal/mol) and dissociation constant (M) of spike RBD-ACE2 docked complexes were obtained from the Prodigy website (https://rascar.science.uu.nl/prodigy/; accessed on August 5, 2024) [21]. In the current study, glycans were omitted from the structural models used for docking and molecular dynamics simulations.

### Molecular dynamics simulation

For checking docked ligand protein complex stability, molecular dynamics (MD) simulation analysis was performed. Schrödinger's Desmond software (version 2019−4) was used to conduct the MD simulations to analyze the stability of the protein-protein complex over 100 nanoseconds in triplicates. The initial complex structures from molecular docking were processed through Schrödinger's Protein Preparation Wizard or Maestro for optimization and energy minimization. System preparation involved the Force field selection of OPLS_2005 for molecular interactions, TIP3P water model in orthorhombic boxes using System Builder as solvation, counterion addition with 0.15M NaCl to simulate physiological conditions for neutralization. The simulation was employed NPT ensemble parameters (i.e., 310K temperature and 1 atm pressure) after initial relaxation phases. Trajectory data was recorded every 100 picoseconds for subsequent analysis [22]. The termini and histidines were protonated based on default settings in Schrödinger’s Protein Preparation Wizard, which typically assigns standard protonation states at physiological pH (~7.0). The pKa or PropKa calculations were not explicitly performed to adjust histidine protonation or local charge states.

## Results

### Multiple sequence alignment, mutations, and 3D structure prediction

**Fig 1:**
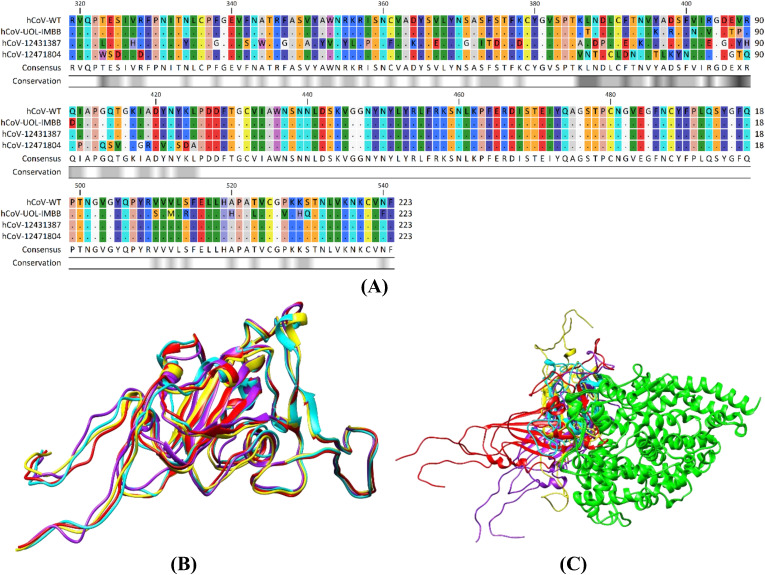
Multiple sequence alignment and predicted structures of RBDs of spike protein. **(A)** Protein sequences from four different variants of SARS-Cov-2 virus was aligned by C-Omega to find out the mutant, conserved and consensus residues. Colored map represents the 0% (black) to 100% (white) residue conservation. **(B)** Superimposed three dimensional computationally predicted structures of wild type and variants. **(C)** Ribbon diagram of superimposed docked complexes of spike protein RBDs of wild and mutant variants. Here, wild type hCoV-WT (red), hCoV-UOL-IMBB (yellow), hCoV-12431387 (cyan), and hCoV-12471804 (purple) variants docked with human ACE2 receptor (green).

The spike RBD of hCoV-WT showed 91.03, 85.65, and 84.75% identity with hCoV-UOL-IMBB, hCoV-12431387, and hCoV-12471804, respectively. All predicted mutation sites retrieved from GISAID database are given in Table 1. InterPro online tool revealed the position of receptor binding domain within the spike protein sequences (from amino acids 319−541). Interestingly, a receptor binding domain (InterPro ID: IPR018548) of total 223 residues started at the amino acid 319 and ends at the residue 541 in all the variants (Fig 1A). The protein sequences of the wild and mutated hCoV-19 variants were used to the predict the three-dimensional (3D) structures of RBD via AlphaFold2 on ColabFold online platform. The predicted 3D structures of all variants, when superimposed, showed clear overall alignment (i.e., 100% query coverage) with the hCoV-WT structure (Fig 1B). The Ramachandran plots were also generated to verify the quality of the predicted 3D models (S1 Fig in S1 File).

**Table 1:** Predicted mutations in hCoV-UOL-IMBB, hCoV-12431387, and hCoV-12471804 variants retrieved from GISAID database.

Variant	hCoV-UOL-IMBB	hCoV-12431387	hCoV-12471804
AC No.	EPI_ISL_5411332	EPI_ISL_15316452	EPI_ISL_15316448
Mutations	L387T	T323L	T323W
	Y396K	V327H	E324S
	D398R	N334Y	S325D
	V401N	F338G	R328D
	R403V	F342S	K386V
	E406T	A344W	L387N
	V407P	F347G	N388T
	Q409D	V350A	L390C
	V510S	A352Y	C391L
	V512M	W353V	F392D
	S514R	R355Y	T393N
	A520H	K356L	Y396T
	T523L	I358P	A397L
	P527V	C361F	D398K
	K529H	D364K	S399Y
	S530Q	V367E	F400N
	N540F	S371G	E406G
		S373I	V407T
		F374T	R408Q
		S375D	I410P
		K378D	G413Q
		K386A	Q414S
		N388D	T415V
		D389P	I418G
		F392E	A419R
		N394K	Y421V
		R403E	Y423S
		D405G	K424D
		V407Y	L425A
		R408H	

### Molecular docking of spike receptor binding domain with human ACE2 receptor

**Fig 2:**
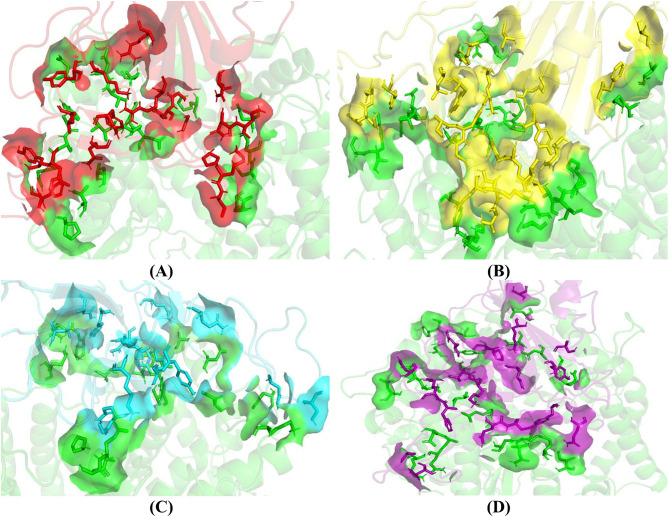
Molecular interaction of spike RBD variants with human ACE2 receptor proteins. **(A)** Interacting residues and 3D binding pocket of RBD of wild type hCoV-WT (red) spike protein with human ACE2 receptor (green). **(B)** Interacting residues and 3D binding pocket of RBD of variant hCoV-UOL-IMBB (yellow) spike protein with human ACE2 receptor (green). **(C)** Interacting residues of RBD and 3D binding pocket of variant hCoV-12431387 (cyan) spike protein with human ACE2 receptor (green). **(D)** Interacting residues and 3D binding pocket of RBD of hCoV-12471804 (purple) spike protein with human ACE2 receptor (green).

Molecular docking predicts the interactions and binding patterns between different protein-ligand, protein-DNA and/or protein-protein molecules. HADDOCK web server was employed for protein-protein docking of human ACE2 receptor with RBDs of wild and Pakistani SARS-CoV-2 variants (Fig 2). The overall HADDOCK score was predicted by Root Mean Square Deviation (RMSD), Van der Waals, electrostatics, desolvation, and restraints violation energy of the docked complex. The hCoV-12431387 variant showed high HADDOCK dock score (i.e., 224.8 ± 32.0), while hCoV-12471804 and hCoV-UOL-IMBB exhibited low and comparable scores to the wild type, respectively (Table 2). The mutation in amino acid residues in receptor binding domain alter the molecular interactions such as hydrogen bonding, hydrophobic interaction, and salt bridges of RBD with human ACE2 receptor (Fig 2). For instance, the mutated R403V, Q409D, G413Q, and Y421V did not show hydrogen bond interactions predicted in the wild type. In contrast, S375D, R408Q, I410P, and G413Q represented novel hydrogen bonding in hCoV-12471804 variant as reported in Table S4 in S1 File. Similarly, mutated G413Q, T415V, Y421V, and S373I developed novel hydrophobic interaction in hCoV-12431387, and hCoV-12471804 variants (Table S2 and Table S3 in S1 File, respectively). Only hCoV-UOL-IMBB variant showed three salt bridges through residues Arg408, Lys417, and Lys444 with wild non-mutated variant. However, the mutated V407P and Q409D reduced the number of salt bridges of residue Arg408 in hCoV-UOL-IMBB (Table S2 in S1 File). In hCoV-12431387 and in hCoV-12471804 mutated variants, the Arg408 lost the salt bridge formation after mutations of R408H and R408Q, respectively (Table S3 and S4 in S1 File). The outcome of these interactions showed mutational changes in RBD of spike protein could alter the binding affinity of these two proteins.

**Table 2:** HADDOCK docking scores of human ACE2 receptor with spike RBD of wild and Pakistani SARS-CoV-2 variants (averaged over three independent HADDOCK runs).

Parameters	hCoV-WT	hCoV-UOL-IMBB	hCoV-12431387	hCoV-12471804
HADDOCK Score	195.7 ± 12.4	183.1 ± 10.4	224.8 ± 32.0	139.6 ± 21.8
Cluster Size	15	14	6	12
RMSD	8.3 ± 0.4	1.3 ± 1.0	0.5 ± 0.3	0.7 ± 0.6
Van der Waals Energy	−102.3 ± 5.0	−102.1 ± 3.1	−95.5 ± 9.6	−128.4 ± 3.0
Electrostatic Energy	−252.8 ± 30.4	−276.5 ± 34.3	−162.8 ± 40.9	−226.2 ± 25.8
Desolvation Energy	−22.6 ± 6.1	−10.0 ± 5.2	−23.9 ± 2.7	−20.3 ± 4.0
Restraints Violation energy	3711.9 ± 120.2	3505.5 ± 133.3	3767.7 ± 288.3	3335.2 ± 150.8
Buried Surface Area	3116.1 ± 87.7	3647.7 ± 109.1	3172.9 ± 150.5	4066.4 ± 55.9
Z-Score	−2.0	−1.9	−1.1	−2.1

### Mutations in spike receptor binding domain improved interaction with ACE2 receptor

**Fig 3:**
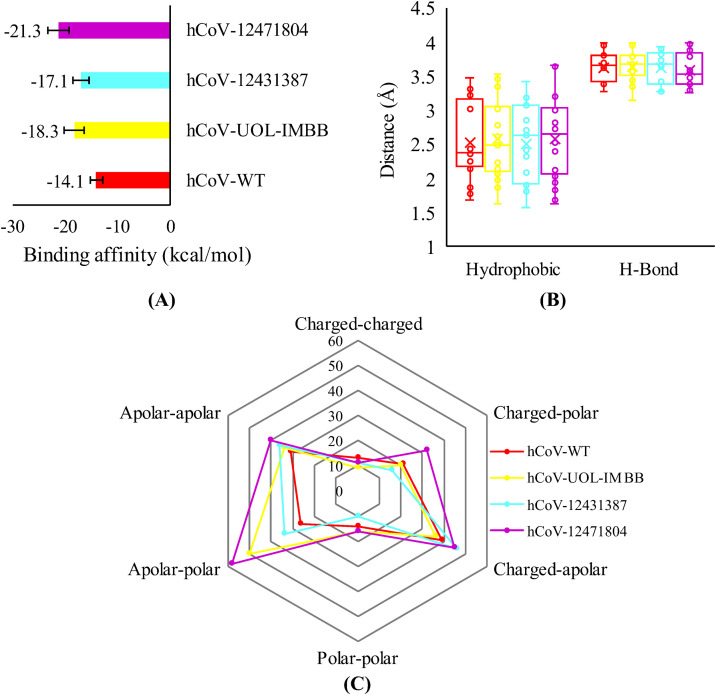
Influence of mutations in the spike receptor binding domain from interactions with the ACE2 receptor. **(A)** Predicted binding affinity (ΔG) (kcal/mol) between spike RBDs and hACE2 docked complexes via Prodigy web-based server. Bars represent mean and error bars show standard error of three independent docked complexes. **(B)** Influence on bond distance (Å) of hydrogen bonding and hydrophobic interrelations between spike RBDs and hACE2 docked complexes. **(C)** The number of possible contacts predicted between the spike RBDs and hACE2 docked complexes via Prodigy web-based server.

To further confirm the binding affinity (ΔG) (kcal/mol) and dissociation constant (M) of spike RBD-ACE2 docked complexes (pdb) obtained from HADDOCK, the Prodigy web server was utilized. Remarkably, hCoV-12471804 represented the lowest ΔG value (−21.3 kcal/mol) compared to the wild type (−14.1 kcal/mol) representing improved binding affinity with hACE2 (Fig 3A, Table 3). It is essential to note that the absolute binding affinity values (kcal/mol generated by PRODIGY are predictive and derived from a model, not direct experimental measurements. Therefore, the results are interpreted comparatively across four modeled variants, rather than as definitive, absolute dissociation constants. PRODIGY reports a Root Mean Square Error (RMSE) of approximately 1.89 kcal/mol when benchmarked against experimental data, which serves as an approximate error range, or confidence interval, for the predicted binding affinity (ΔG).

**Table 3:** An overview of the effect of mutations in different Pakistani variants to binding with human ACE2 receptor compared to the non-mutated wild spike protein.

Parameters	hCoV-WT	hCoV-UOL-IMBB	hCoV-12431387	hCoV-12471804
No. of intermolecular contacts	144	165	153	202
No. of charged-charged contacts	13	9	11	11
No. of charged-polar contacts	21	20	16	32
No. of charged-apolar contacts	39	36	46	45
No. of polar-polar contacts	14	16	10	16
No. of apolar-polar contacts	26	50	34	58
No. of apolar-apolar contacts	31	34	36	40
Percentage of apolar NIS residues	35.19	36.19	35.95	35.66
Percentage of charged NIS residues	25.78	25.70	25.21	25.35
Predicted binding affinity (kcal/mol)	−14.1	−18.3	−17.1	−21.3
Predicted dissociation constant (M) at 37°C	1.2e-10	1.3e-13	8.2e-13	9.0e-16

Moreover, the predicted bond distances between interacting residues varied in all the variants and have been shown in Fig 3B. This might be due to the mutated residues, loss of interactions of mutated residues, and formation of novel interactions found in the binding pocket of the RBD of the spike protein with hACE2. The number of binding interactions has also been influenced by the mutations in the critical residues of the spike RBDs. The variants hCoV-UOL-IMBB, hCoV-12431387, and hCoV-12471804 were predicted with 165, 153, and 202 of total interactions, respectively, which is greatly higher than the wild type (144) (Table 3). The hCoV-12471804 was predicted with the highest number of interactions (202) including charged-polar contacts (32), apolar-polar contacts (58), and apolar-apolar contacts (40), which is greatly high (Fig 3C). These results proved the improved protein-protein interactions between spike RBDs of Pakistani variants with hACE2.

### Molecular dynamics simulation

**Fig 4:**
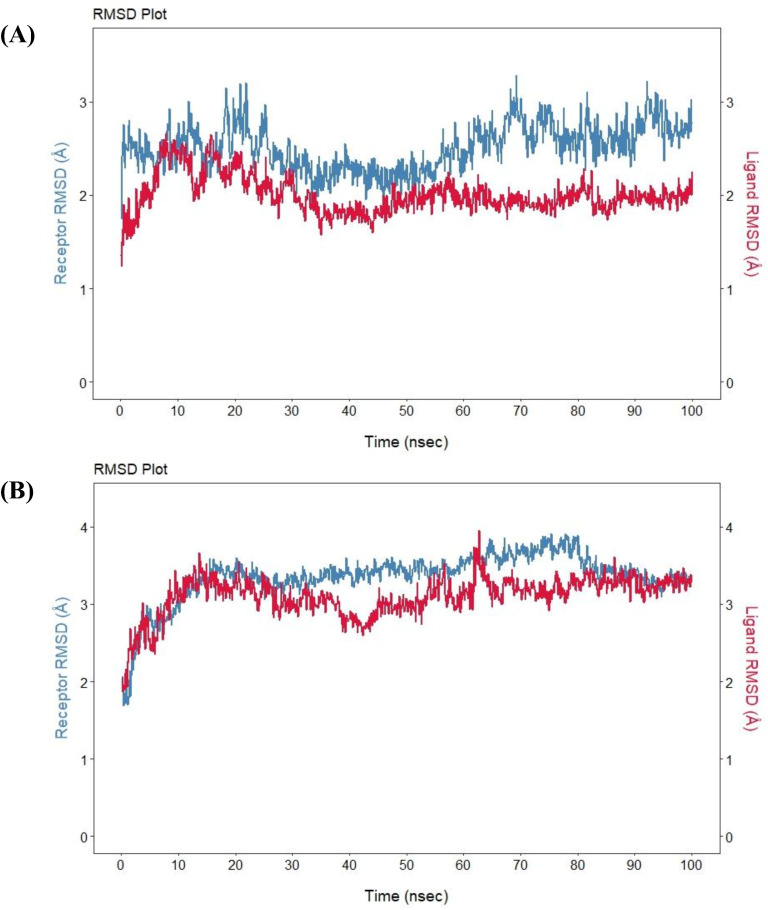
Root Mean Square Deviation (RMSD) plots of. (A) hACE2 and hCoV-WT complex, (B) hACE2 and hCoV-12471804 complex.

The stability of human ACE2 (hACE2) receptor protein with spike RBD of wild (hCoV-WT) and a mutant hCoV-12471804 was checked through molecular dynamics simulation as shown in Fig 4. The RMSD plot of hACE2 and hCoV-WT complex demonstrated that the complex was found to be stable over 100 ns simulation time as shown in Fig 4A. The RMSD of the hACE2 was started from ~1.5 Å and reached 2.5–3.0 Å rapidly within the first 10 ns. Afterwards, the RMSD value fluctuated between 2–3.2 Å for the rest of the simulation time with no signs of returning to the initial level. These minor fluctuations showed that hACE2 protein underwent some conformational changes and did not show signs of unfolding or instability. The RMSD of the hCoV-WT (ligand) was also observed to be increased rapidly in the first few nanoseconds and reached a highest value of 2.0–2.5 Å. The RMSD of hCoV-WT was stabilized and fluctuated between 1.5–2.2 Å, which showed that hCoV-WT maintained a relatively stable conformation or binding mode throughout the simulation time. The RMSD value of the hCoV-WT was lower compared to the hACE2, which is showing that hCoV-WT was more conformationally stable or tightly bound within the binding pocket of the receptor protein.

Similarly, the RMSD of hACE2 and hCoV-12471804 is also characteristic of a stable protein-protein complex where the target protein maintained structural integrity (Fig 4B). The hACE2 showed a characteristic or typical equilibrium pattern of MD simulation. The RMSD value starting from 1.8 Å increased rapidly during initial simulation time of 15 nanoseconds and reached 3.5 Å. Some moderate fluctuations were observed in the RMSD between 20–40 ns where the value was found to be dropped to ~3.0 Å following the recovery. After 40 ns, the protein was stabilized at 3.5–3.8 Å, which showed that the protein was reached a relatively stable conformation for the rest of the simulation time. The mutant protein hCoV-12471804 (i.e., ligand) exhibited more dynamic behavior throughout simulation time. The RMSD of the ligand starting from 2.0 Å increased rapidly to ~3.5 Å within the first 10 ns. Unlike hACE2 protein, hCoV-12471804 showed more fluctuations throughout the simulation time with overall a stable binding with the receptor protein. The MD simulation was run in triplicates and the RMSD graphs of second MD simulation run have been given in S2 Fig in S1 File.

The time evolution of hydrogen bonds during a molecular dynamics simulation of hCoV-12471804 complex in shown in S3 Fig in S1 File. For hACE2 and hCoV-WT complex, at the start, there was some variability in hydrogen bond count ranging from about 8–16 (S3a Fig in S1 File). Between 10,000 ps and 40,000 ps, the number of H-bonds generally declined, reaching a lower range of roughly 7–10. After 40,000 ps, the count fluctuated but showed a modest rising trend, stabilizing around 10–13 hydrogen bonds. The fluctuations observed correspond to the natural dynamics and conformational flexibility during the MD simulation. Similarly, for hACE2 and hCoV-12471804 complex, the subsequent increase and stabilization in hydrogen bond count could reflect the system reaching equilibrium, where stable intra- or inter-molecular interactions were formed (S3b Fig in S1 File). Fluctuations seen in each time window are typical of thermal motion and conformational transitions in biological molecules.

**Fig 5:**
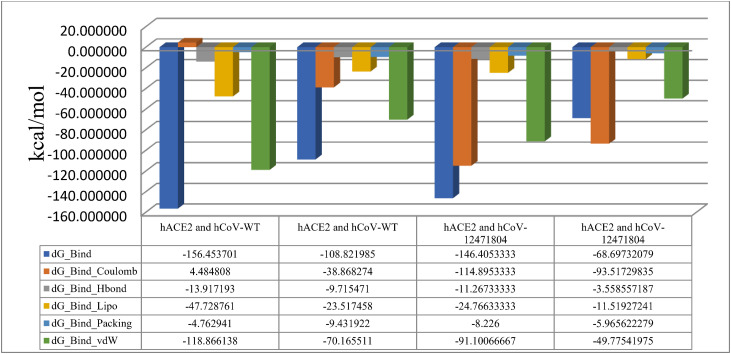
Molecular mechanics generalized born surface area (MM-GBSA) of hACE2 and hCoV-WT complex, and hACE2 and hCoV-12471804 complex.

The molecular mechanics generalized born surface area (MM-GBSA) was also calculated for the MD simulation as displayed in Fig 5. For hACE2 and hCoV-WT complex, the blue bars (i.e., ΔGBind) were found to be consistently negative across all time points and ranged from −108 to −156 kcal/mol. These negative values showed that hCoV-WT remained firmly bound to the receptor throughout the simulation with energetically favorable binding at all sampled times. The green bars (i.e., vdW) were also found to be strongly negative, which revealed that van der Waals interactions would be the major stabilizing force in the binding. The contributions of vdW were stable across the simulation time, which supported the idea of a well-packed binding interface. For hACE2 and hCoV-WT complex, the average ΔG was found to be −156.45, the ΔG range was −156.78 to −94.82, and the standard deviation was determined to 29.35. Similarly, in case of hACE2 and hCoV-12471804 complex, the total binding energy (i.e., ΔGBind) was found to be consistently favorable across all time points (i.e., typically below −60 kcal/mol), which is indicating a stable binding through the simulation time. The mutant hCoV-12471804 (i.e., ligand) also maintained a strong and stable binding to the hACE2 protein throughout the simulation, which was predominantly driven by different forces including van der Waals and electrostatic interactions, with supportive hydrophobic interactions. The effects of hydrogen bonding and packing played their role as minor contributors. The profile of hACE2 and hCoV-12471804 is a characteristic of a strongly bound complex with stable dynamics over the course of MD simulation. For hACE2 and hCoV-12471804 complex, the average ΔG was calculated to be −69.66 kcal/mol with ΔG range of −144.07 to −61.48 and the standard deviation of 36.44.

## Discussion

Coronaviruses are positively-stranded RNA enveloped viruses with remarkable mutation rate to evolve during the transmission [23]. The spike protein of SARS-CoV-2 virus helps the virus to interact with angiotensin-converting enzyme 2 (ACE2) of human which facilitates the entry of virus into human body [24].

The current study was conducted to reveal the effect of mutations in RBD of spike protein on the binding affinity of three Pakistani variants to hACE2. Among 1236 spike mutated sequences from Pakistan, three variants with high mutation rate in RBD region were retrieved from GISAID database (Table 1) and aligned by ClustalW as shown in Fig 1 [16]. The 3D structures of mutated spike proteins were predicted by ColabFold2 based on reported mutations given in Figs 1 and 2 and structure quality of the predicted models were analyzed by Ramachandran plot as shown in S1 Fig in S1 File. Previous studies have reported that mutations in the receptor binding domain of spike protein can facilitate the viral entry and enable immune escape [12,25,26].

Several mutations in spike protein have been reported throughout the pandemic which can increase the transmissibility of coronavirus [27]. The first mutation in spike protein was found to be D614G which was reported in Europe in March 2020 and then quickly spread with 100% prevalence [28]. Studies have shown that this mutation delays the separation of spike S1 and S2 subunits after binding with ACE2 and enhances the density of intact spike trimers on the surface of the virus [29,30]. Later, another mutation N501Y was observed in many lineages inside the receptor binding domain which ultimately improved the affinity of spike protein for ACE2 [31].

In this study, protein-protein interactions were explored using the HADDOCK server, with docking performed in three independent runs for each protein complex and different parameters such as cluster size, cluster RMSD values, Van der Waals energy, electrostatic energy, desolation energy, restraints violation energy, buried surface area, and Z-score were recorded and given in Fig 2 and Table 2. The quality of the protein model was measured by Z-score as well as deviation of total energy of the structure in relation to energy distribution resulting from random conformations [32]. In HADDOCK docking analysis, a lower Z-score (−2.1) was recorded in hCoV-12471804-ACE docking compared to other mutated variants as well as wild non-mutated variant. The score indicates that the mutations occur in this variant improved the binding and enhanced the complex stability [33]. Remarkably, hCoV-12471804 represented the lowest ΔG value (−21.3 kcal/mol) compared to wild (−14.1 kcal/mol) and other mutated variants which showed that mutations in this variant had enhanced the binding potential of the mutated spike protein with human ACE2 receptor.

The RBD of SARS-CoV-2 have some critical residues including Gly446, Tyr449, Leu455, Phe486, Tyr491, Gln493, Gly496, Gln498, Thr500, Asn501, and Gly502 [34]. Among these, Phe486, Gln493, and Asn501 of RBD are most important residues because they assist the hACE2 to recognize the receptor binding domain of the spike protein [35]. Moreover, Ala475 and Phe484 residues play key role to neutralize the antibodies and escape from body immunity [36]. Meanwhile, Lys31 and Lys353 residues of hACE2 help to stabilize the spike-hACE2 complex by providing significant amount of energy [37].

Results of protein-protein interaction showed that only two mutations in hCoV-UOL-IMBB variant (i.e., R403V and Q409D) are present in wild non-mutated RBD and participate in interaction with hACE2 through hydrogen bonding with Val364 and Asp295 residues (Table S1 and S2 in S1 File). Only hCoV-UOL-IMBB variant showed three common salt bridges with wild type (S2 Table in S1 File). Precisely, in non-mutated wild variant, at position 408 arginine was present which was involved in interaction with hACE2 receptor through a salt bridge but in hCoV-12471804 variant, arginine was replaced by glutamine which made interaction with hACE2 residue (Asn149) through hydrogen bonding which escalated the complex stability. Results showed that the three key mutations (i.e., I410P, G413Q, and Y421V) in hCoV-12471804 variant are involved in interactions with hACE2 as reported in Table S4 in S1 File. Molecular docking of mutated hCoV-12471804 variant results showed that Pro410 interacted with Asn154 residue of hACE2 by conventional hydrogen bonding, where Gln413 made hydrophobic interaction with Tyr279 residue of hACE2. Moreover, Val421 interacted with Val364 residue through conventional hydrogen bonding as shown in Table S4 in S1 File. In the hCoV-12471804 variant, only one mutation (R328D) was observed in the β-sheet region of the RBD, and this substitution mutation does not alter the β-sheet structure (S4 Fig in S1 File). Structural analysis of the hCoV-12471804 variant indicates that despite the observed mutations, the overall RBD structure remains conserved, retaining its native fold and compatibility with ACE2 binding (Fig S5 Figure). As a result of these mutations, the interactions between mutated RBD and hACE2 become stronger and stable which is proved by simulations outcomes.

Similarly, three amino acid residues in non-mutated spike RBD including Arg403, Asp405, and Arg408 are involved in hydrogen bonding, hydrophobic interaction, and salt bridge, respectively are mutated in hCoV-12431387 variant. In the mutated variant at position 403, arginine was substituted by glutamic acid, whereas at position 405 aspartic acid was replaced by glycine, and at position 408 arginine was replaced by histidine. The docking results showed that Glu403 displayed conventional hydrogen bonding with Val364 residue of hACE2 (S3 Table in S1 File) and played important role in the stability of the binding pose. Overall, the combined effect of substitution mutations in RBD resulted in enhanced binding efficiency with hACE2 [37]. Previous studies have reported that mutations in the RBD amino acids K417, E484, L452, T478, and N501 significantly increased the affinity of spike protein for hACE2 [38–40].

Hydrophobic integrations and hydrogen bonding are the key factors to study protein-protein interactions [41]. While the PRODIGY calculations provided a useful pre-simulation comparison of the relative binding trends between the wild-type and mutant complexes, the MM-GBSA energies derived from full molecular dynamics trajectories represent the actual and system-specific interaction energies of the complexes under simulated physiological conditions. The HADDOCK results indicate that the mutated variants have higher affinity towards the hACE2 compared to the hCoV-wild variant. The higher affinity between mutated RBD and hACE2 is one of the key factors which enhance the viral transmission and infection rate [42]. Same results have been reported in the previous studies where the presence of mutation in receptor binding domain of spike protein increased the binding affinity with hACE2 [43–46]. These mutations in RBD of spike protein play fundamental role in the viral immune evasion. Moreover, neutralizing antibodies actively target the RBD and inhibit the viral infection. The identified mutations are located in or near antibody escape and neutralizing epitope regions, which could alter local structure, residue exposure, or charge, potentially reducing antibody binding and neutralization [11,41]. However, this study primarily assessed their effect on hACE2 binding, so direct impacts on antibody interactions were not evaluated, highlighting a limitation that requires further experimental validation. Therefore, it is essential to track any mutation in RBD to understand viral escape from immune system and its evolution.

In the current study, glycans were omitted from the structural models used for docking and molecular dynamics simulations, which is a limitation. N343 glycosylation on the spike RBD and multiple glycosylation sites on ACE2 (e.g., N90 and N322) are known from experimental studies to influence binding affinity and conformational dynamics. Literature shows glycans at these sites can both strengthen and hinder ACE2-spike interactions (e.g., by steric shielding or stabilizing contacts) [36]. Previous study reported that the mutations at T478 and E484 positions of RBD facilitate the virus to escape from antibody-mediated neutralization process [47]. Such mutations can challenge the mode of action of antiviral drugs and medications and need more attention. To design the antiviral drugs and vaccines, it is more important to carefully studies such mutations in target sites in receptor binding domain as well as on other target sites of the spike protein. Epidemiological data from Pakistan are limited, so the impact of individual mutations or variants on transmissibility and outbreaks can only be inferred. Our computational results suggest mutations may enhance spike–ACE2 binding and infectivity, but direct links to population-level outcomes require further genomic, clinical, and experimental studies.

## Conclusion

The development of a safe and effective vaccine or therapeutic drug is very necessary to end the pandemic and stop the transmission of coronavirus in the future. In this study, we assessed the structural variation as a result of mutations in the spike protein especially in the receptor binding domain that affect the binding with human ACE2 using *in silico* methods including protein-protein docking and molecular dynamic simulation. The outcome of this study will provide an understanding of the dynamic interaction between spike protein and hACE2. The results of protein-protein interaction showed that mutated hCoV-12471804 variant has higher number of interactions (202), lower Z-score (−2.1), increased buried surface area (4066.4 ± 55.9), and high binding affinity (−21.3 kcal/mol) with hACE2 compared to hCoV-wild variant. The improved interaction between mutated hCoV-variants and hACE2 is due to the accumulative effect of substitution mutations in RBD of the spike protein which can assist the entry of the virus into the human body. Exploring and defining the effect of these mutations on interaction with human ACE2 helps to understand why newly arose SARS-CoV-2 strains spread more quickly and increased the severity of the disease. This study could assist to understand the role of mutations in the RBD of spike protein on viral entry into the human body and help researchers to design appropriate vaccines and therapeutics against mutated viral variants. It is important to acknowledge that the findings presented in this study are based exclusively on computational (*in silico*) methods, including structure prediction, molecular docking, and binding affinity calculations. Definitive confirmation of these *in silico* predictions will require further experimental validation to quantify the kinetic and thermodynamic parameters of the hACE2-RBD interaction.

## Supporting information

S1 File S1 Table. Interacting Residues of RBD of spike wild type (hCoV-WT) and Human ACE2 receptor proteins. **S2 Table.** Interacting Residues of Spike hCoV-UOL-IMBB and Human ACE2 receptor proteins. **S3 Table**. Interacting Residues of Spike hCoV-12431387 and Human ACE2 receptor proteins. **S4 Table**. Interacting Residues of Spike hCoV-12471804 and Human ACE2 receptor proteins. **S1 Fig**: Ramachandran plots to verify the quality of predicted structures. **S2 Fig**. RMSD plots of (A) hACE2 and hCoV-WT complex, (B) hACE2 and hCoV-12471804 complex. **S3 Fig**. Number of hydrogen bonds over time during the molecular dynamics simulation of 100 ns. (a) hACE2 and hCoV-WT complex, (b) hACE2 and hCoV-12471804 complex. **S4 Fig.** Comparison of the β-sheet region of the RBD in the wild-type (red) and hCoV-12471804 mutant variant (purple). Table Comparison of the overall RBD structure of the wild-type (red) and hCoV-12471804 mutant variant (purple).
(DOCX)
