# Supplementary material for: Computational prediction of binding affinity and structural impact of three Pakistani SARS-CoV-2 spike RBD variants on human ACE2 interaction
Source: PLoS One. 2026 Apr 1;21(4):e0346242. doi: 10.1371/journal.pone.0346242 (PMC13042742; doi:10.1371/journal.pone.0346242)
Supplement: S1 File — Interacting Residues of RBD of spike wild type (hCoV-WT) and Human ACE2 receptor proteins. S2 Table. Interacting Residues of Spike hCoV-UOL-IMBB and Human ACE2 receptor proteins. S3 Table. Interacting Residues of Spike hCoV-12431387 and Human ACE2 receptor proteins. S4 Table. Interacting Residues of Spike hCoV-12471804 and Human ACE2 receptor proteins. S1 Fig: Ramachandran plots to verify the quality of predicted structures. S2 Fig. RMSD plots of (A) hACE2 and hCoV-WT complex, (B) hACE2 and hCoV-12471804 complex. S3 Fig. Number of hydrogen bonds over time during the molecular dynamics simulation of 100 ns. (a) hACE2 and hCoV-WT complex, (b) hACE2 and hCoV-12471804 complex. S4 Fig. Comparison of the β-sheet region of the RBD in the wild-type (red) and hCoV-12471804 mutant variant (purple). Table Comparison of the overall RBD structure of the wild-type (red) and hCoV-12471804 mutant variant (purple). (DOCX) [file pone.0346242.s001.docx]

**Computational Prediction of Binding Affinity and Structural Impact of Three Pakistani SARS-CoV-2 Spike RBD Variants on Human ACE2 Interaction**

**Muhammad Usama ^1†^, Muhammad Azeem ^2†^, Ghulam Mustafa ^1*^**

^1^Department of Biochemistry, Government College University Faisalabad, Faisalabad 38000 Pakistan

^2^College of Life Sciences, Anhui Normal University, Wuhu 241000, China

*Corresponding author’s Email: [drghulammustafa@gcuf.edu.pk](mailto:drghulammustafa@gcuf.edu.pk)

# Supplementary Data

| (A) | (B) |
| --- | --- |
| 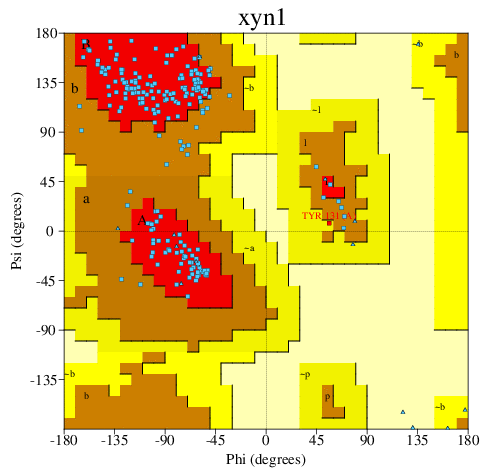 | 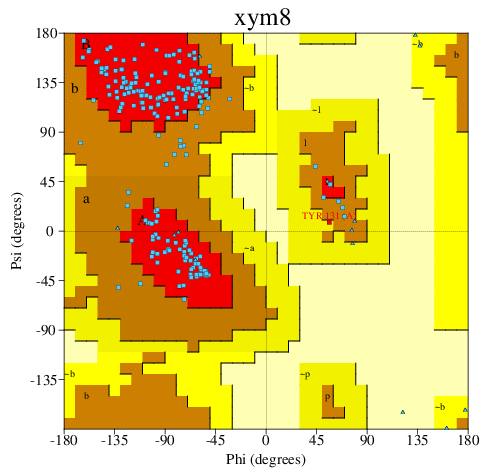 |
| (C) | (D) |
| 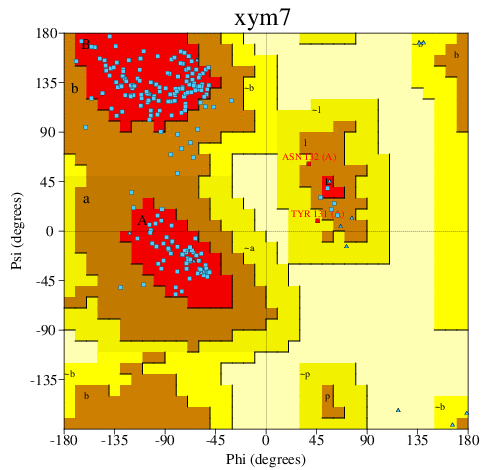 | 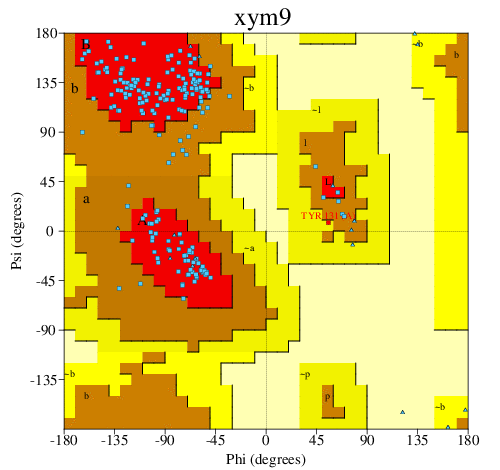 |

**Figure S1**. Ramachandran plots to verify the quality of predicted structures.

| 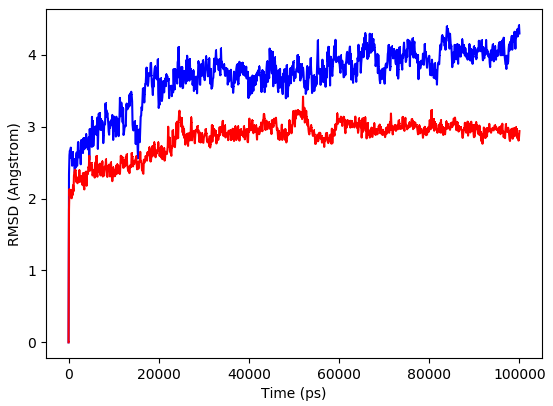 | 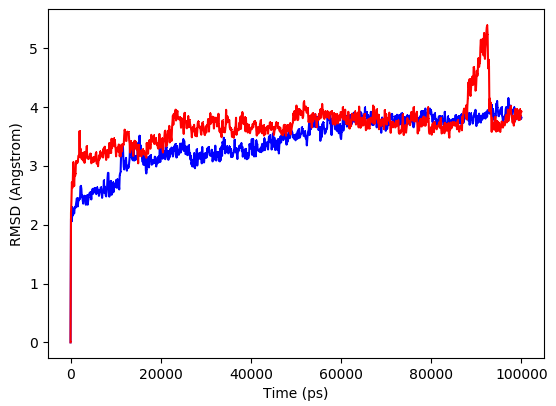 |
| --- | --- |
| **(a)** | **(b)** |

**Figure S2**. RMSD plots of (A) hACE2 and hCoV-WT complex, (B) hACE2 and hCoV-12471804 complex.

| 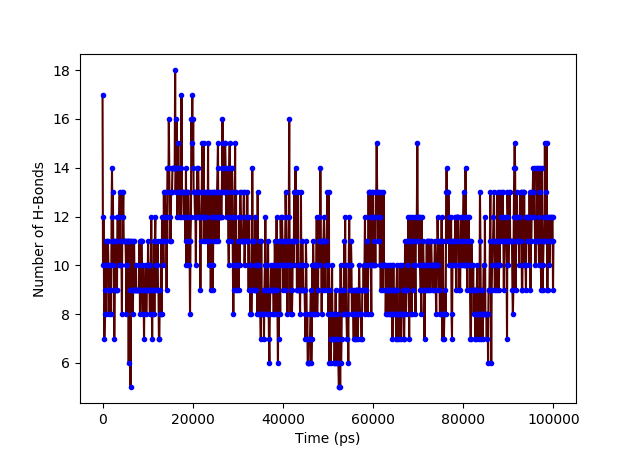 |
| --- |
| **(a)** |
| 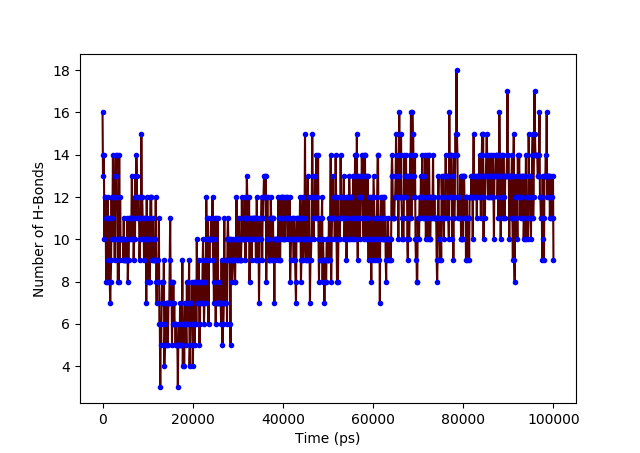 |
| **(b)** |

**Figure S3**. Number of hydrogen bonds over time during the molecular dynamics simulation of 100 ns. (a) hACE2 and hCoV-WT complex, (b) hACE2 and hCoV-12471804 complex.


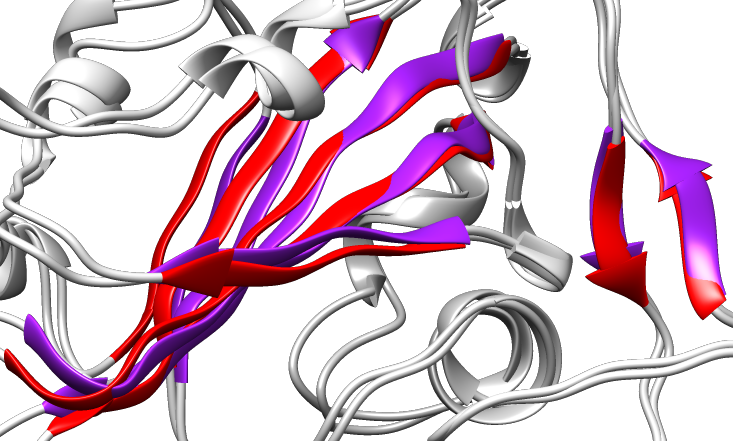


**Figure S4**. Comparison of the β-sheet region of the RBD in the wild-type (red) and hCoV-12471804 mutant variant (purple).


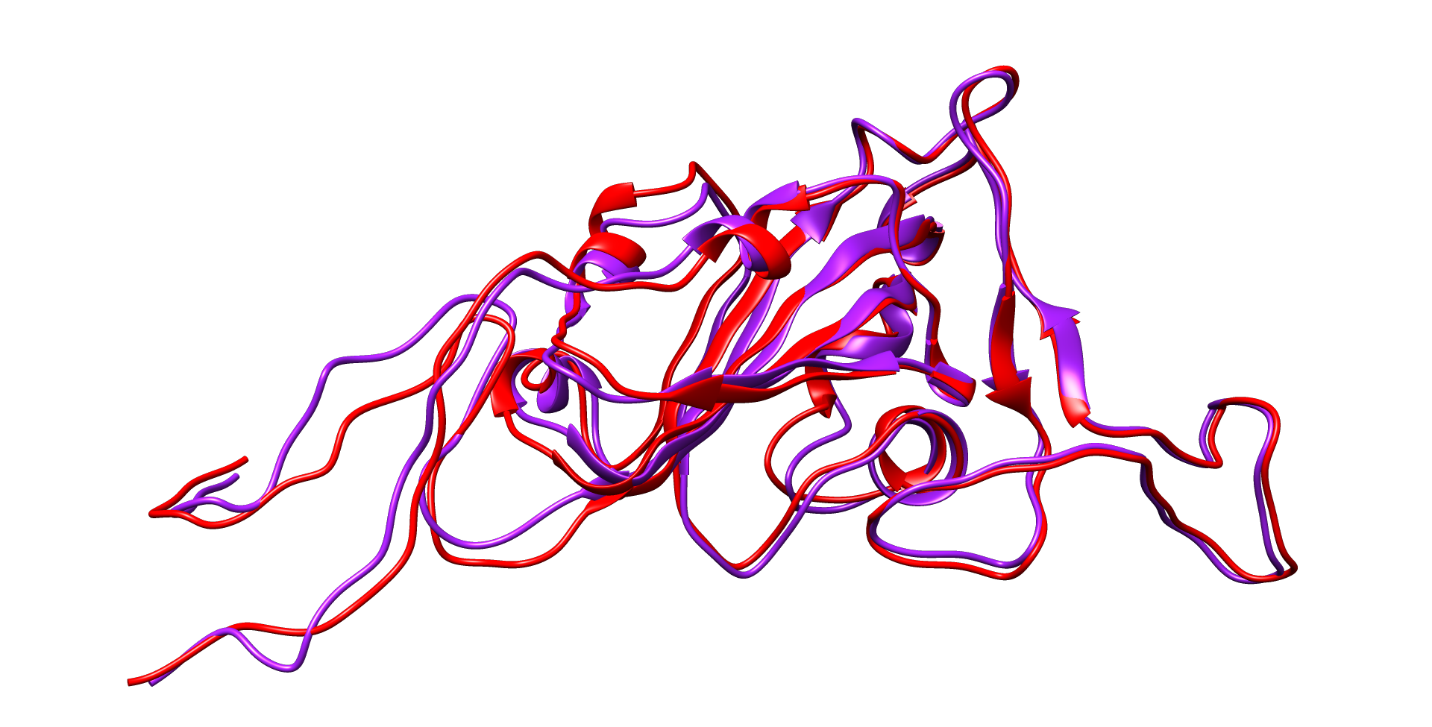


**Figure S5**. Comparison of the overall RBD structure of the wild-type (red) and hCoV-12471804 mutant variant (purple).

**Table S1**. Interacting Residues of RBD of spike wild type (hCoV-WT) and Human ACE2 receptor proteins.

| **Spike Residue** | **ACE2 Residue** | **Distance** |
| --- | --- | --- |
| Hydrophobic Interactions | | |
| Asp405 | Asp292 | 3.87 |
| Lys417 | Val298 | 3.4 |
| Tyr421 | Val298 | 3.81 |
| Val445 | Glu145 | 3.71 |
| Tyr453 | Val364 | 3.62 |
| Leu455 | Val364 | 3.95 |
| Phe456 | Asp303 | 3.99 |
| Phe456 | Ala301 | 3.27 |
| Tyr489 | Gln305 | 3.69 |
| Tyr489 | Asp303 | 3.7 |
| Gln493 | Val364 | 3.66 |
| Pro499 | Pro146 | 3.42 |
| Val503 | Tyr279 | 3.43 |
| Val503 | Tyr279 | 3.42 |
| Tyr505 | Met366 | 3.38 |
| Hydrogen Bond | | |
| Arg403 | Val364 | 2.35 |
| Arg403 | Val364 | 3.02 |
| Gln409 | Asp295 | 3.21 |
| Lys417 | Asp295 | 1.85 |
| Tyr421 | Asp299 | 1.67 |
| Asn437 | Asn154 | 2.38 |
| Asn439 | Glu150 | 1.76 |
| Val445 | Glu145 | 2.29 |
| Tyr453 | Thr365 | 3.32 |
| Glu484 | Thr334 | 2.36 |
| Glu484 | Asp335 | 3.3 |
| Gly485 | Gln305 | 2.24 |
| Gln493 | Thr362 | 2.43 |
| Gln493 | Val364 | 2.37 |
| Pro499 | Asn149 | 3.47 |
| Gly502 | Asp367 | 2.14 |
| Salt Bridge | | |
| Arg408 | Asp292 | 4.25 |
| Arg408 | Asp295 | 4.59 |
| Lys417 | Asp292 | 5.12 |
| Lys444 | Glu150 | 5.17 |

**Table S2**. Interacting Residues of Spike hCoV-UOL-IMBB and Human ACE2 receptor proteins

| **Spike Residue** | **ACE2 Residue** | **Distance** |
| --- | --- | --- |
| Hydrophobic Interactions | | |
| Tyr369 | Asn338 | 3.81 |
| Pro384 | Val339 | 3.56 |
| Thr385 | Val339 | 3.78 |
| Asp405 | Pro146 | 3.51 |
| Lys417 | Glu150 | 3.68 |
| Leu455 | Leu156 | 3.8 |
| Phe456 | Asp157 | 3.15 |
| Glu484 | Leu156 | 3.51 |
| Phe486 | Tyr255 | 3.83 |
| Phe486 | Pro253 | 3.95 |
| Phe486 | Tyr255 | 3.56 |
| Tyr489 | Asp157 | 3.44 |
| Tyr489 | Leu156 | 3.68 |
| Gln493 | Leu156 | 3.98 |
| Pro499 | Asp292 | 3.49 |
| Thr500 | Phe438 | 3.73 |
| Val503 | Asp367 | 3.34 |
| Hydrogen Bond | | |
| Arg408 | Gly147 | 3.53 |
| Arg408 | Gly147 | 3.41 |
| Thr415 | Cys141 | 2.83 |
| Tyr421 | Asn134 | 2.61 |
| Tyr421 | Glu140 | 1.62 |
| Tyr421 | Glu140 | 2 |
| Asn437 | Thr365 | 2.62 |
| Asn437 | Thr365 | 2.23 |
| Asn439 | Thr365 | 2.23 |
| Lys444 | Thr294 | 3.02 |
| Tyr453 | Asn154 | 2.26 |
| Asn460 | Asn137 | 3.05 |
| Tyr473 | Asn159 | 3.35 |
| Tyr487 | Asp157 | 3.48 |
| Tyr489 | Tyr255 | 3.05 |
| Tyr489 | Tyr255 | 2.09 |
| Tyr489 | Tyr158 | 2.48 |
| Tyr494 | Asp157 | 2.14 |
| Tyr498 | Asp157 | 2.13 |
| Ser499 | Asn154 | 2.06 |
| Gln500 | Asn290 | 1.97 |
| Pro503 | Thr294 | 3.46 |
| Tyr505 | Lys441 | 1.88 |
| Tyr505 | Asp367 | 2.2 |
| Tyr506 | Asn149 | 1.85 |
| Tyr318 | Pro146 | 2.78 |
| Gln319 | Asp367 | 3.07 |
| Salt Bridge | | |
| Arg408 | Glu145 | 4.06 |
| Lys417 | Glu150 | 3.46 |
| Lys444 | Asp295 | 3.6 |

**Table S3**. Interacting Residues of Spike hCoV-12431387 and Human ACE2 receptor proteins

| **Spike Residue** | **ACE2 Residue** | **Distance** |
| --- | --- | --- |
| Hydrophobic Interactions | | |
| Ile373 | Val339 | 3.27 |
| Glu406 | Lys363 | 3.9 |
| Lys417 | Thr294 | 3.64 |
| Asp427 | Glu150 | 3.41 |
| Asp427 | Ile151 | 3.68 |
| Tyr453 | Val298 | 3.93 |
| Leu455 | Thr294 | 3.66 |
| Leu455 | Asp295 | 3.73 |
| Phe456 | Asp292 | 3.74 |
| Tyr473 | Pro289 | 3.27 |
| Ala475 | Pro289 | 3.48 |
| Tyr489 | Asp295 | 3.93 |
| Tyr505 | Ala304 | 3.82 |
| Tyr505 | Val364 | 3.32 |
| Hydrogen Bond | | |
| Asp375 | Lys341 | 1.92 |
| Asp375 | Lys341 | 1.56 |
| Asp375 | Asn338 | 1.97 |
| Glu403 | Val364 | 1.9 |
| Gly413 | Asn149 | 3.42 |
| Gly413 | Glu150 | 1.84 |
| Gln414 | Glu150 | 2.62 |
| Lys417 | Thr365 | 2.86 |
| Tyr421 | Asp292 | 2.08 |
| Tyr421 | Asp292 | 2.7 |
| Asp427 | Ile151 | 3.18 |
| Asn437 | Gly337 | 2.85 |
| Ser477 | Lys288 | 2.4 |
| Asn487 | Gln429 | 3.11 |
| Tyr489 | Asp292 | 3.22 |
| Gln493 | Asp295 | 1.8 |
| Gly502 | Asp303 | 3.06 |
| Gly504 | Thr334 | 2.31 |
| Tyr508 | Gly337 | 2.66 |
| Salt Bridge | | |
| Lys424 | Glu150 | 2.67 |

**Table S4**. Interacting Residues of Spike hCoV-12471804 and Human ACE2 receptor proteins

| **Spike Residue** | **ACE2 Residue** | **Distance** |
| --- | --- | --- |
| Hydrophobic Interactions | | |
| Thr376 | Glu150 | 3.41 |
| Lys378 | Asn154 | 3.58 |
| Gln413 | Tyr279 | 3.48 |
| Val415 | Met366 | 3.61 |
| Val421 | Val364 | 3.78 |
| Tyr453 | Lys363 | 3.97 |
| Leu455 | Lys363 | 3.78 |
| Leu455 | Thr334 | 3.38 |
| Phe456 | Val364 | 3.29 |
| Phe456 | Asp303 | 3.39 |
| Arg457 | Val298 | 3.25 |
| Tyr473 | Asp303 | 3.28 |
| Ala475 | Gln305 | 3.97 |
| Phe486 | Trp328 | 3.87 |
| Asn487 | Gln305 | 3.98 |
| Val503 | Glu145 | 3.39 |
| Hydrogen Bond | | |
| Lys378 | Asn154 | 2.81 |
| Ser383 | Leu156 | 2.57 |
| Gln408 | Asn149 | 3.04 |
| Pro410 | Asn154 | 2.51 |
| Gln413 | Phe285 | 1.82 |
| Gln413 | Asn437 | 1.93 |
| Gln413 | Tyr279 | 2.02 |
| Gln413 | Lys441 | 2.18 |
| Leu455 | Val364 | 3 |
| Arg457 | Asp295 | 2.83 |
| Asn460 | Asp295 | 2.11 |
| Asn460 | Asp299 | 1.67 |
| Gln474 | Asp303 | 3.66 |
| Asn487 | Gln305 | 2.73 |
| Tyr489 | Leu333 | 3.21 |
| Gln493 | Asp335 | 2.06 |
| Ser494 | Asn338 | 2.71 |
| Ser494 | Lys341 | 3.04 |
| Ser494 | Asp335 | 3.19 |
| Tyr505 | His345 | 2.39 |
| Tyr505 | Cys344 | 3.63 |
| Tyr508 | Glu145 | 1.61 |
| Salt Bridge | | |
| Lys378 | Glu150 | 3.18 |
